# A method for nonlinear electric-thermal coupling calculations of bushings based on unbiased gradient-free smooth domains

**DOI:** 10.1371/journal.pone.0297750

**Published:** 2024-04-16

**Authors:** Yuhui Feng, Zhongqing Yang, Chao Gao, Erya Gao, Ruixiao Meng

**Affiliations:** 1 China Nuclear Power Operations Company Limited, Shenzhen, China; 2 Suzhou Nuclear Power Research Institute Company Limited, Suzhou, China; 3 National Engineering Research Center for Nuclear Power Plant Safety & Reliability, Suzhou, China; TU Dublin Blanchardstown Campus: Technological University Dublin - Blanchardstown Campus, IRELAND

## Abstract

High-voltage dry-type bushings, serving as the crucial junctions in DC power transmission, represent equipment with the highest failure rate on the DC primary side, underscoring the critical importance of monitoring their condition. Presently, numerical simulation methods are commonly employed to assess the internal state of bushings. However, due to limitations in the efficiency of multi-physics field computations, the guidance provided by numerical simulation results in the field of power equipment condition assessment is relatively weak. This paper focuses on solving the electrical-thermal coupling in high-voltage dry-type bushings. Addressing the most widely used tetrahedral mesh in numerical computations, we propose an efficient solution method based on the concept of "smooth domains." This method involves partitioning the volume centroids of the elements into multiple smooth domains within the computational domain. Electric and thermal conduction matrix calculations occur within these smooth domains, rather than within the grid or element interiors. This approach eliminates the need for traditional element mapping and complex volume integration. To demonstrate the effectiveness of this method, we use high-voltage dry-type bushings as a case study, comparing the performance of our approach with traditional finite element algorithms. We verify the algorithm’s computational efficiency and apply it to the analysis of typical temperature anomalies in bushings, further illustrating its suitability for electrical equipment condition assessment.

## Introduction

High-voltage direct current (HVDC) transmission is an important means for long-distance inter-regional power transmission due to its high transmission capacity, long-distance capability, low cost, and good control performance [1]. HVDC bushings, as the "throat" of the AC-DC hybrid grid, bear the electrical connections of the entire voltage and current of the system. However, HVDC dry-type bushings frequently experience faults, accounting for 16.7% of DC primary equipment failures and having the highest frequency of causing converter valve lockouts. Therefore, advanced sensing technology and information-based methods are needed for monitoring the status of the bushings [2]. HVDC dry-type bushings have a compact insulation structure primarily using SF6 gas as the main insulation medium, making it impossible to install sensors internally [3]. Currently, online monitoring methods for bushings mainly rely on parameters such as gas pressure, discharge, leakage current, and dielectric loss, which can only provide external data. The diagnosis of the bushing’s operational status often depends on power outage experiments, which suffer from insufficient monitoring parameters and criteria [4, 5]. Conducting multi-physics field coupling analysis of bushings can obtain internal state parameters, providing new criteria for analyzing the bushing’s operational status. Currently, the main causes of bushing failures are largely attributed to electrical and thermal faults, such as insulation breakdown [6, 7] and material aging [8]. Finite element analysis with electrical-thermal coupling is the most commonly used method for addressing these issues.

The conventional finite element method (CFEM) discretizes the solution domain into multiple elements using a mesh and solves for variables such as electric potential and temperature at each node within these elements [9]. Since Gaussian quadrature is commonly used as the main integration method in CFEM, the mesh elements need to be transformed into tetrahedral shapes through geometric mapping. Subsequently, the integration involves applying the corresponding number of Gaussian points for each element, making the integration process complex and time-consuming. As the scale of physical problems increases and the number of mesh elements grows, the computational efficiency of traditional finite element methods gradually decreases [10].

Current approaches to improving numerical computation efficiency primarily focus on enhancing the numerical algorithms themselves. For instance, starting from polyhedral meshes and establishing mathematical models for convective heat transfer that adapt to various boundary conditions has been employed to enhance the efficiency of heat transfer simulations [11]. Combining the meshless Galerkin method with electromagnetic simulations has simplified solving boundary value problems and improved the efficiency of electromagnetic field simulations, particularly for handling interfaces between different media [12]. In recent years, the use of artificial intelligence models as solvers, employing data-driven approaches for numerical computations, and leveraging the capabilities of high-performance computing units have been explored to enhance computational efficiency [13].

The Smoothed Finite Element Method (SFEM) is a representative high-precision numerical model that improves upon the finite element method. It discretizes control equations by partitioning smooth domains, simplifies the integration process using methods such as Lagrange approximation, reduces the demands on grid quality, and exhibits characteristics such as high computational accuracy, strong adaptability of grid elements, and high computational efficiency [14–17].

This paper is based on the theory of Smoothed Finite Elements and presents an unbiased gradient-free electric-thermal coupled body Smoothed Finite Element Method. It provides a discretization approach for electric-thermal coupled equations based on smooth domains and develops a gradient-free nonlinear electric-thermal coupled solver. Finally, using high-voltage dry-type bushings as an example, this paper employs the electric-thermal coupled computational model proposed herein to investigate the impact of typical operating conditions on bushing heating. This research provides new insights to advance the application of electric-thermal coupled simulation in the field of bushing condition assessment.

## Derivation of the electro-thermal coupling equation for high-voltage dry-type bushings

### Electro-thermal coupling control equations and boundary conditions

The governing equations for electrical conduction and thermal conduction are represented as shown in Eq (1).

$$\left\{\begin{array}{l} \nabla \cdot J=0\\ \nabla \cdot q=q_{J} \end{array}\right.$$ (1)

Where J is current density, A/m^2^, *q*_*J*_ is thermal load, W/m^3^.

In this paper, only the Joule heating generated by the electric field is considered as the heat source, and the formula for calculating Joule heating *q*_*J*_ is given in Eq (2).


$$q_{J}=E\cdot J$$ (2)

For the three-dimensional steady-state problem, based on the weighted residual method (Galerkin method), we multiply both sides of the equation by a test function and integrate. Combining this with the Green’s formula, we obtain the weak form of the governing equation for control, as shown in Eq (3).


$$\left\{\begin{array}{l} \int_{\Omega }\nabla N^{T}\cdot \sigma \cdot \nabla \varphi d\Omega -\int_{\Gamma }N\cdot \sigma \cdot \nabla \varphi \cdot \vec{n}d\Gamma =0\\ \int_{\Omega }\nabla N^{T}\cdot \lambda \cdot \nabla \theta d\Omega =\int_{\Omega }N\cdot q_{J}d\Omega +\int_{\Gamma }N\cdot q_{n}d\Gamma \end{array}\right.$$ (3)

In the equation, where:

N represents the shape functions (test functions).

σ is the electrical conductivity, measured in siemens per meter (S/m).

$\vec{n}$ is the outward normal vector to the boundary of the solution domain.

*q*_*n*_ represents the inward normal heat flux (W/m^2^).

Ω represents the solution domain.

Γ represents the boundary of the solution domain.

To solve the equation shown in Eq (3), it is necessary to specify boundary conditions. The electro-thermal coupling problem studied in this paper primarily involves three types of boundary conditions: Dirichlet, Neumann, and Robin boundary conditions.

Dirichlet boundary conditions primarily involve specifying fixed electric potential and fixed temperature boundaries, as shown in Eq (4).


$$\left\{\begin{array}{l} \varphi =\varphi_{s}\\ T=T_{s} \end{array}\right.$$ (4)

In the equation, *φ*_*s*_ and *T*_*s*_ represent the fixed electric potential and fixed temperature, respectively. Since the conjugate gradient method requires the matrix to be symmetric positive definite, it is essential to ensure that the imposition of essential boundary conditions does not break the symmetry of the matrix.

Neumann boundary conditions are primarily used to specify the outward normal direction derivatives on the boundary. These conditions typically involve the normal current density and heat flux, as shown in the Eq (5).

$$\left\{\begin{array}{c} -\sigma \nabla \varphi \cdot (-n)=J_{s}\\ -\lambda \nabla \theta \cdot (-n)=q_{s} \end{array}\right.$$ (5)

*J*_*s*_ represents the specified inward normal current density, and *q*_*s*_ represents the specified inward normal heat flux.

Robin boundary conditions are a combination of function values and the outward normal direction derivatives on the boundary. In the context of electro-thermal coupling problems, these conditions primarily describe convective heat transfer, as shown in the Eq (6).


$$-k\nabla \theta \cdot (-n)=h(\theta_{ext}-\theta )$$ (6)

In which, *h* represents the convective heat transfer coefficient in W/(m^2^·K), and *T*_*ext*_ represents the specified external temperature.

## Discretization method for electro-thermal coupling equations in traditional finite element analysis

The CFEM (Conventional Finite Element Method) typically discretizes the solution domain into multiple elements using a mesh and fits the physical field variables within each element using the values at mesh nodes. The interpolation forms for the electric potential and temperature within the elements are shown in Eq (7) [18].


$$\begin{array}{l} \varphi =\sum_{i=1}^{ne}N_{i}\cdot \varphi_{i}\\ \theta =\sum_{i=1}^{ne}N_{i}\cdot \theta_{i} \end{array}$$ (7)

In the equation, φ_i_ and θ_i_ are the discrete values of electric potential and temperature at the ith node of the element, respectively. ne represents the number of nodes in the element. Ni represents the shape functions at each node position. For three-dimensional problems, the shape functions used are as shown in Eq (8):

$$\begin{array}{l} N_{1}(x,y,z)=(1-x-y-z)\\ N_{2}(x,y,z)=x\\ N_{3}(x,y,z)=y\\ N_{4}(x,y,z)=z \end{array}$$ (8)

**Fig 1:**
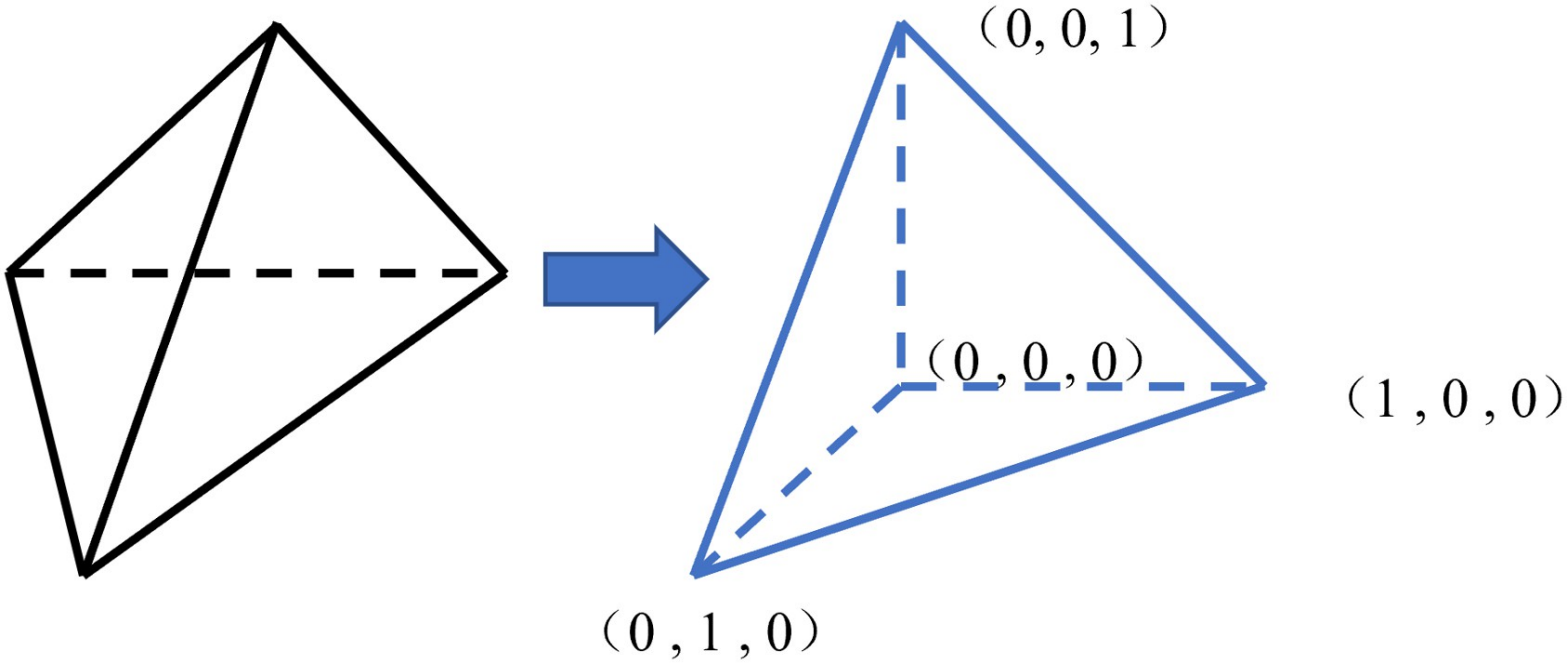
Schematic diagram of mesh element mapping.

CFEM based on the discrete elements mentioned above, requires mapping grid elements into right-angled tetrahedra with an edge length of 1 when solving Eq (3) using Gaussian integration, as shown in Fig 1. By employing Gaussian points with a weight of 1 at [0.577, -0.577] for Eq (3), you can calculate the electric potential *φ* and temperature *θ* at various grid nodes.

### Discretization of electro-thermal coupling using smoothed finite element method

The SFEM algorithm involves redividing the mesh into multiple smoothed domains and approximating gradients as constant matrices using Lagrange interpolation. This simplifies the integration process and reduces the computational time associated with Gaussian integration.

**Fig 2:**
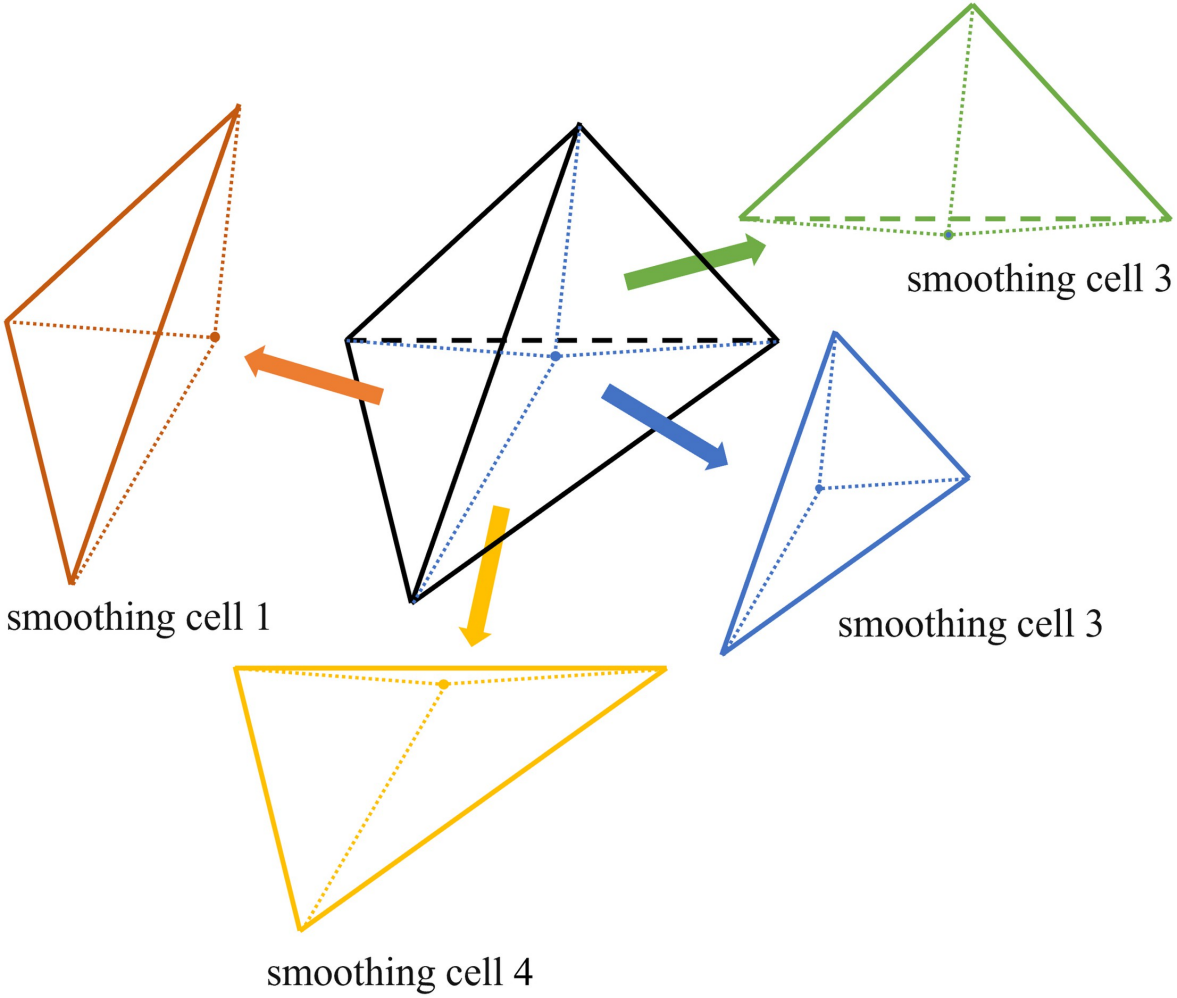
Grid cell smoothing domain division.


In this study, smoothed domains are partitioned using the mesh element’s centroid as the reference point. For three-dimensional grids, the grid nodes are connected to the element’s centroid, forming smoothed domains along with the grid edges, as illustrated in Fig 2. Each smoothed domain satisfies Eq (9).

$$\begin{array}{l} \Omega =\Omega_{1}\cup \Omega_{2}\cup \Omega_{3}\ldots \cup \Omega_{ns}\\ \begin{array}{cc} \Omega_{i}\cap \Omega_{j}=\emptyset & i\neq j \end{array} \end{array}$$ (9)

In the equation, ns represents the number of smoothed domains.

Smoothing the gradients within each smoothed domain, using shape function N as an example, is expressed as shown in Eq (10).


$$B_{\Omega i}\left(x_{C}\right)=\int_{\Omega i}\nabla N(x)\Phi \left(x-x_{C}\right)\text{d}\Omega$$ (10)

According to the integration by parts method, the right-hand side of the equation as show in Eq (11).


$$\begin{array}{l} B_{\Omega i}\left(x_{C}\right)=\int_{\Gamma }N(x)n\left(x\right)\Phi \left(x-x_{C}\right)\text{d}\Gamma \\ -\int_{\Omega }N(x)\nabla \Phi \left(x-x_{C}\right)\text{d}\Omega \end{array}$$ (11)

In the equation, *W* represents the smoothing function. In reference [17], a smoothing function is defined as follows:

$$\Phi \left(x-x_{C}\right)=\left\{\begin{array}{ll} 1/A_{C} & x\in \Omega_{C}\\ 0 & x\notin \Omega_{C} \end{array}\right.$$ (12)

Where $AC=\int_{\Omega_{C}}d\Omega$, and *Ω*_*C*_ is the smoothing domain. Substituting Eq (12) into Eq (11) yields the following formula:

$$B_{\Omega i}\left(x_{C}\right)=\frac{1}{A_{C}}\int_{\Gamma_{C}}N(x)n\left(x\right)\text{d}\Gamma$$ (13)

In the equation, ΓC represents the boundary of the smoothed domain, and n is the outward normal to the boundary of the smoothed domain.

When this integral can be approximated using a single Gaussian point, it can be transformed into Eq (14).


$$B_{\Omega i}\left(x_{C}\right)=\sum_{i=1}^{M}N(x_{i}^{GP})n_{i}^{C}S_{i}^{C}$$ (14)

In the equation, $N(x_{i}^{GP})$ represents the values of the shape functions at the midpoint of the interface, and their areas correspond to $S_{i}^{C}$ and $n_{i}^{C}$ respectively, with respect to the unit normal of the element.

It can be observed that SFEM decomposes the gradients and computes them using boundary integrals, eliminating the need for differentiation operations.

Applying the above gradient approximation method based on smoothed domains to the electro-thermal coupling equation, all the gradients of shape functions in Eq (3) are replaced with the form of Eq (14), transforming the integration into a sum of integrations over each smoothed domain, as shown below.


$$\left\{\begin{array}{l} \int_{\Omega }\nabla N^{T}\cdot \sigma \cdot \nabla Nd\Omega =\sum_{i=1}^{M}B_{\Omega_{i}}^{T}\sigma B_{\Omega_{i}}A_{\Omega_{i}}\\ \int_{\Omega }\nabla N^{T}\cdot \lambda \cdot \nabla Nd\Omega =\sum_{i=1}^{M}B_{\Omega_{i}}^{T}\lambda B_{\Omega_{i}}A_{\Omega_{i}} \end{array}\right.$$ (15)

## Unbiased electro-thermal coupling smoothed finite element computational method

### Unbiased smoothed finite element computational method

**Fig 3:**
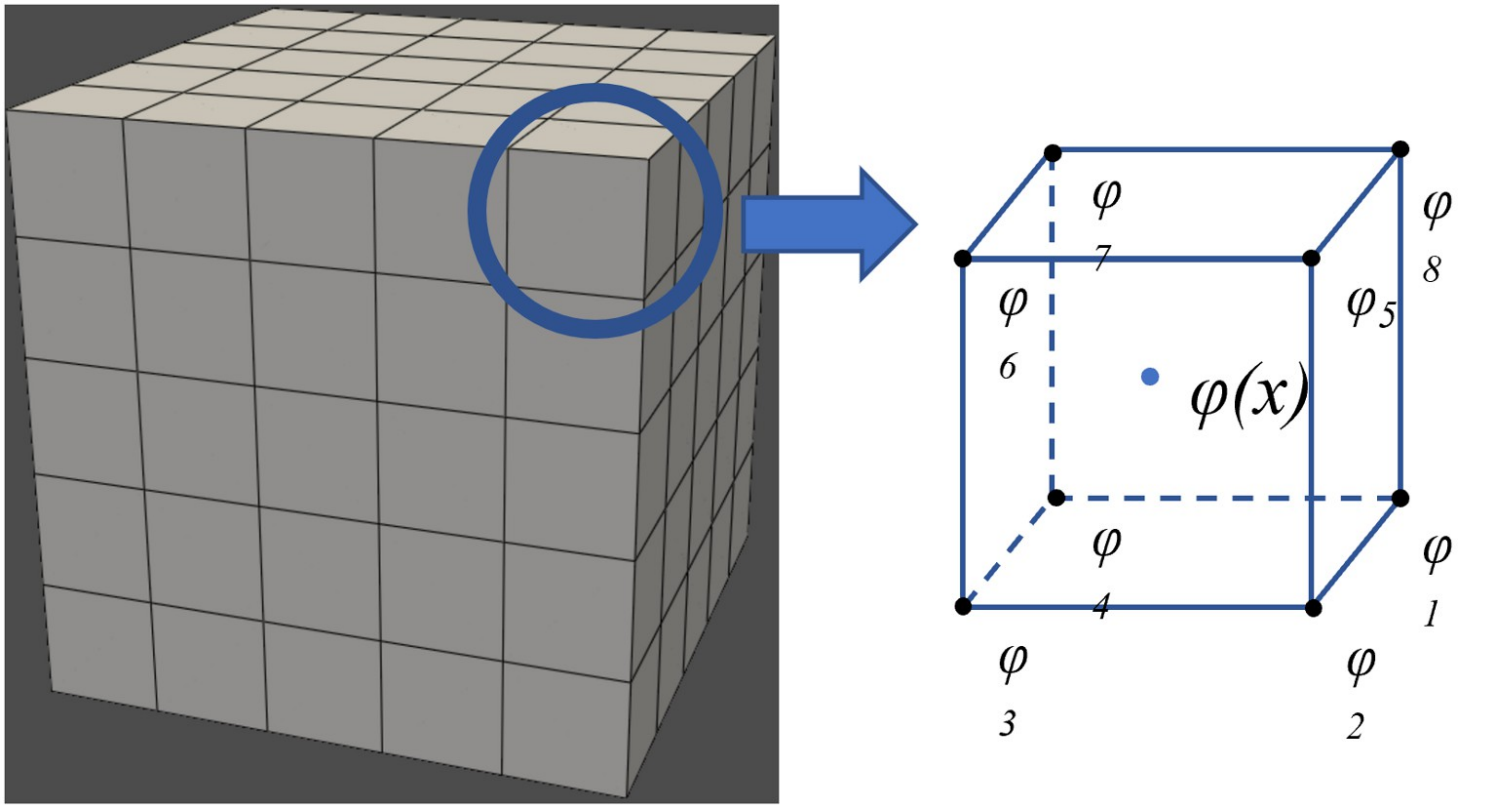
Grid division example.

According to the definition of shape functions, the value at any point within the grid is equal to the sum of the products of the values at the grid nodes and the values of the shape functions at that point. An illustration of the element nodes is shown in Fig 3.

By substituting Eq (7) into Eq (3), the electro-thermal conduction equation can be expressed in the following form:

$$\begin{array}{l} \int_{\Omega }\nabla N^{T}\cdot \sigma \cdot \nabla Nd\Omega \vec{\varphi }=\int_{\Gamma }N\cdot \sigma \cdot \nabla N\cdot \vec{n}d\Gamma \vec{\varphi }\\ \int_{\Omega }\nabla N^{T}\cdot \lambda \cdot \nabla Nd\Omega \vec{\theta }-\int_{\Gamma }N\cdot \lambda \cdot \nabla \theta \cdot \vec{n}d\Gamma =\int_{\Omega }N\cdot Qd\Omega \end{array}$$ (16)

Expressing the above equation in matrix form:

$$\left[\begin{array}{cc} K^{\varphi } & 0\\ 0 & K^{\theta }+K_{2}^{\theta } \end{array}\right]\cdot \left[\begin{array}{c} \varphi \\ \theta \end{array}\right]=\left[\begin{array}{c} I\\ Q^{J}\text{+}Q^{S} \end{array}\right]$$ (17)

In the formula, the electric conduction matrix *K*^*φ*^ *=* ∫_Ω_ ∇*N*^*T*^ · *σ* · ∇*Nd*Ω,

Heat conduction matrix is *K*^*θ*^ *=* ∫_Ω_ ∇*N*^*T*^ · *λ* ∇*Nd*Ω,

External current load vector is $I=\int_{\Gamma }N\cdot \sigma \cdot \bar{J}\cdot \vec{n}d\Gamma$, $\bar{J}$ is the external input current density, given by the boundary conditions,

Joule heat load vector is *Q*^*J*^ = ∫_Ω_ *N* · *Qd*Ω,

Since the convective heat flux is decomposed into $K_{2}^{\theta }$ 和 ***Q***^*S*^, The convective heat transfer boundary is *q* = *h*(*T*-*T*_*1*_), so $K_{2}^{\theta }=\int_{\Gamma }N^{T}\cdot h\cdot Nd\Gamma$, $Q^{S}=\int_{\Gamma }N\cdot T_{1}\cdot hd\Gamma$, *T*_*1*_ is the ambient temperature specified by the boundary conditions, and *h* is the convective heat transfer coefficient.

The materials used in high-voltage dry-type bushings have non-linear relationships between thermal conductivity and electrical conductivity with respect to temperature. Special considerations are required for the electric conductivity matrix *K*^*φ*^, the thermal conductivity matrix *K*_*θ*_, and the current load vector I.

Since this study employs a weakly coupled electro-thermal approach, the electrical conductivity σ in the *K*^*φ*^ matrix can be calculated using the temperature values from the previous iteration. This approach accounts for the temperature-dependent electrical conductivity and allows for an iterative solution process that updates both temperature and electrical conductivity iteratively until convergence is achieved.

Expressing the thermal conductivity matrix *K*^*θ*^ in the following form.


$$\begin{array}{l} K^{\theta }=\sum_{i=1}^{M}\int_{\Omega_{i}}B_{\Omega_{i}}^{T}\lambda \left(\theta \right)B_{\Omega_{i}}d\Omega_{i}\\ =\sum_{i=1}^{M}B_{\Omega_{i}}^{T}\int_{\Omega_{i}}\lambda \left(\theta \right)d\Omega_{i}B_{\Omega_{i}} \end{array}$$ (18)

Within each smoothed domain Ωᵢ, a second-order interpolation is performed using the same shape functions as the integration, as shown in Eq (19).


$$\begin{array}{l} \lambda \left(x\right)=N_{1}\left(x\right)\cdot \lambda_{1}+N_{2}\left(x\right)\cdot \lambda_{2}\\ +N_{3}\left(x\right)\cdot \lambda_{3}+N_{4}\left(x\right)\cdot \lambda_{4} \end{array}$$ (19)

In the equation, N* and λ* represent the shape function and electrical conductivity of the th node, where the value of λ is determined by the function λ(θ). Writing Eq (20) into a matrix form results in the following expression.


$$K^{\theta }=\sum_{i=1}^{M}B_{\Omega_{i}}^{T}\left(\int_{\Omega_{i}}\left[N_{1}\;N_{2}\;N_{3}\;N_{4}\right]d\Omega_{i}\cdot \left[\begin{array}{l} \lambda_{1}\\ \lambda_{2}\\ \lambda_{3}\\ \lambda_{4} \end{array}\right]\right)B_{\Omega_{i}}$$ (20)

Since the integration of the tetrahedral node shape functions is equal to one-fourth of the tetrahedron’s volume, the above equation can be simplified to the form of Eq (21).


$$K^{\theta }=\sum_{i=1}^{M}B_{\Omega_{i}}^{T}\left(\left[\frac{A_{\Omega_{i}}}{4}\;\frac{A_{\Omega_{i}}}{4}\;\frac{A_{\Omega_{i}}}{4}\;\frac{A_{\Omega_{i}}}{4}\right]\cdot \left[\begin{array}{l} \lambda_{1}\\ \lambda_{2}\\ \lambda_{3}\\ \lambda_{4} \end{array}\right]\right)B_{\Omega_{i}}$$ (21)

As a result, it can be observed that this computational method, based on the smooth gradient matrix, does not involve integral operations, leading to a significant improvement in computational speed compared to traditional finite element methods.

Since the current load vector does not involve gradient/partial derivative operations, both can be computed based on the original mesh using traditional finite element methods, and no further elaboration is needed here.

### Program structure

**Fig 4:**
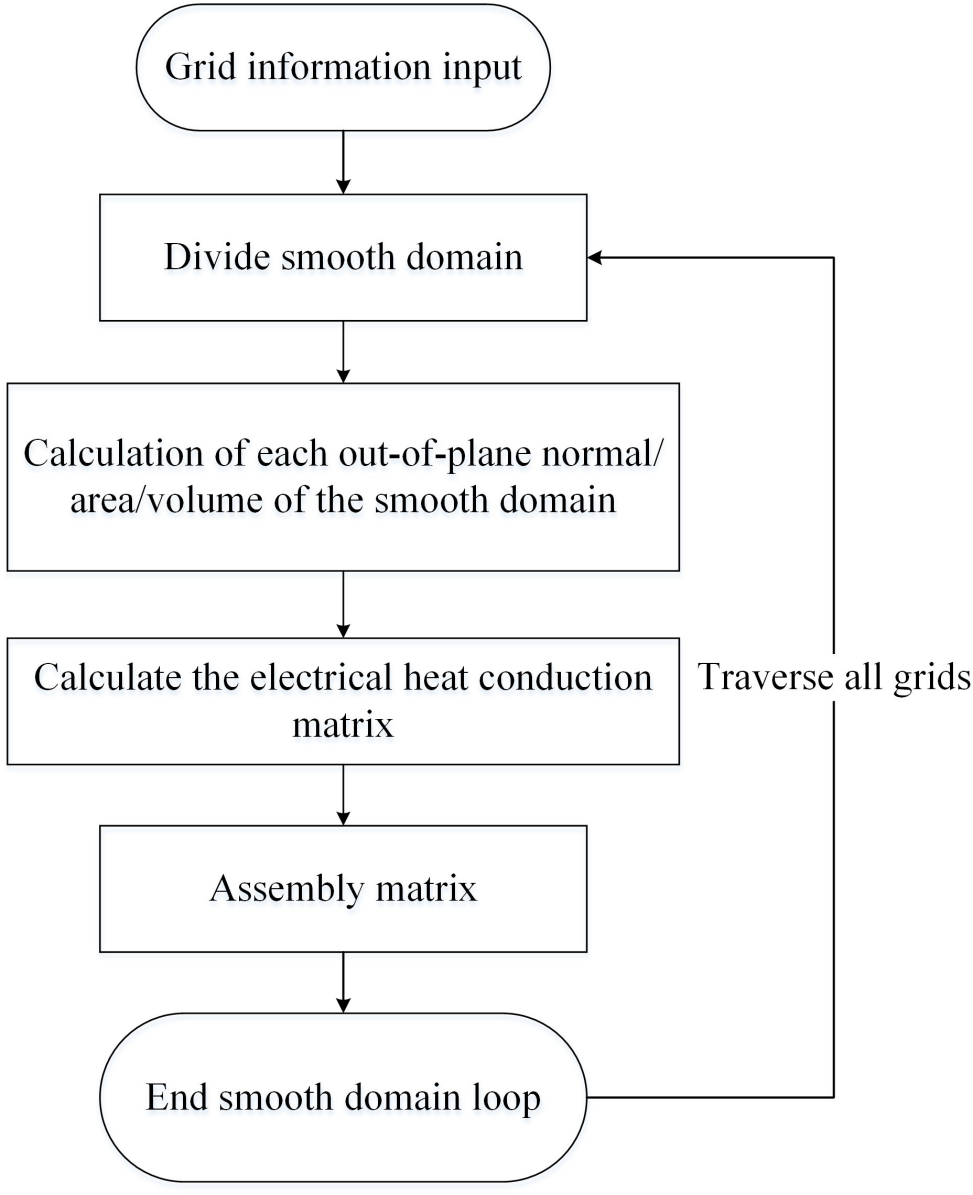
SFEM solution steps.

Fig 4 depicts the steps for nonlinear electro-thermal coupling based on smoothed domains, including grid partitioning, smoothed domain division, computation of smoothed gradient matrices, electro-thermal conduction matrices, setting source excitation, applying natural boundary conditions, calculating electric potential and temperature, and obtaining the system solution through multiple iterations.

The main difference between the improved SFEM and CFEM lies in the approach used to calculate the electric-thermal conduction matrix. In CFEM, this is computed through steps involving element mapping and Gaussian integration. In contrast, the improved SFEM divides grid elements into smooth domains, approximates gradients using Lagrange methods, simplifies the shape function integration through geometric approximation, and iteratively calculates over grid elements.

Both the body-smoothed finite element method and CFEM involve the integration of grid elements. Consequently, their matrix assembly methods are consistent. After completing the matrix calculation assembly, electric potential and temperature values at each grid node are obtained. Since both electrical conductivity and thermal conductivity are functions of temperature, an iterative approach is required. It begins with an initial temperature of *θ* = 20°C, and in each iteration, the temperature from the previous iteration is used to calculate the next iteration’s electrical and thermal conductivities. After multiple iterations, when the relative error between two consecutive iterations is less than 0.001, the result is considered a stable solution, yielding the final solution for the system equations.

## Numerical case

### Bushing electrothermal coupling boundary conditions and material settings

This study establishes a solution method for the nonlinear electro-thermal coupling equation based on the smooth finite element of the body. In this section, high-pressure dry bushing is used as a case to test the performance of the algorithm. Under the same grid conditions, the calculation accuracy and speed of SFEM, commercial CAE software and CFEM are compared. This article uses the julia language to pro-gram, and the Table 1 is the specific con-figuration of the test computer.

**Table 1:** Specifications of the employed computer.

category	information
OS	Windows 10 Professional
CPU	Intel I7-12700H
Number of CPU cores	12
CPU frequency	2.69GHz
CPU RAM	16 GB
GPU	NVIDIA GeForce RTX 4070
Julia Version	1.8.5

**Fig 5:**
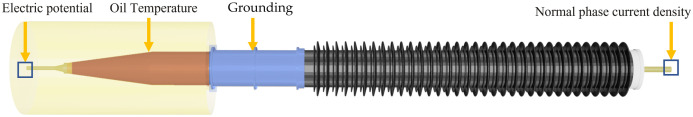
Boundary conditions of dry bushing.

In this paper, the heating conditions of an 400kV dry bushing are simulated under normal operating conditions in a normal temperature environment. The example is chosen to demonstrate the boundary conditions, which are set as follows: the potential is fixed at 432kV, the flange is grounded, the normal phase current is set to 5650A, the ambient temperature is 20°C, and the convective heat transfer coefficient of the outer surface is assumed to be 5W/(m^2^K). Boundary conditions of dry bushing is shown in Fig 5.

In this paper, the material parameters for each component of the bushing are set as follows. The conductivity and thermal conductivity of the conductor and insulation core take into account the nonlinearity of temperature. The material parameters shown in Table 2 are referenced from [19].

**Table 2:** Material parameter setting.

Parameter	Thermal Conductivity W/ (m · K)
Air	0.02
Silicone resin	0.27
SF_6_	0.01
Copper	5.022 × 10^−10^ *T*^4^ − 8.59 × 10^−7^ *T*^3^ + 4.514 × 10^−4^ *T*^2^ − 0.131*T* + 402.1
Flange	44.5
Epoxy Impregnated Paper	−2.01 × 10^−6^ *T*^2^ + 6.6 × 10^−4^ *T* + 0.268
Alloy	0.23*T* + 38.5
parameter	electric conductivity S/m
Air	10^−14^
Silicone resin	5×10^−14^
SF6	1×10^−18^
Copper	** $\frac{1}{1.72\times 10^{-8}\times (1+0.00039\times (T-298))}$ **
Flange	4.3×10^6^
Epoxy Impregnated Paper	** $64.63\times e^{\frac{-12160}{T}}$ **
Alloy	** $\frac{1}{6.619\times 10^{-17}T^{3}-8.193\times 10^{-14}T^{2}+1.451\times 10^{-10}T-1.037\times 10^{-8}}$ **

## Results discussed

**Fig 6:**
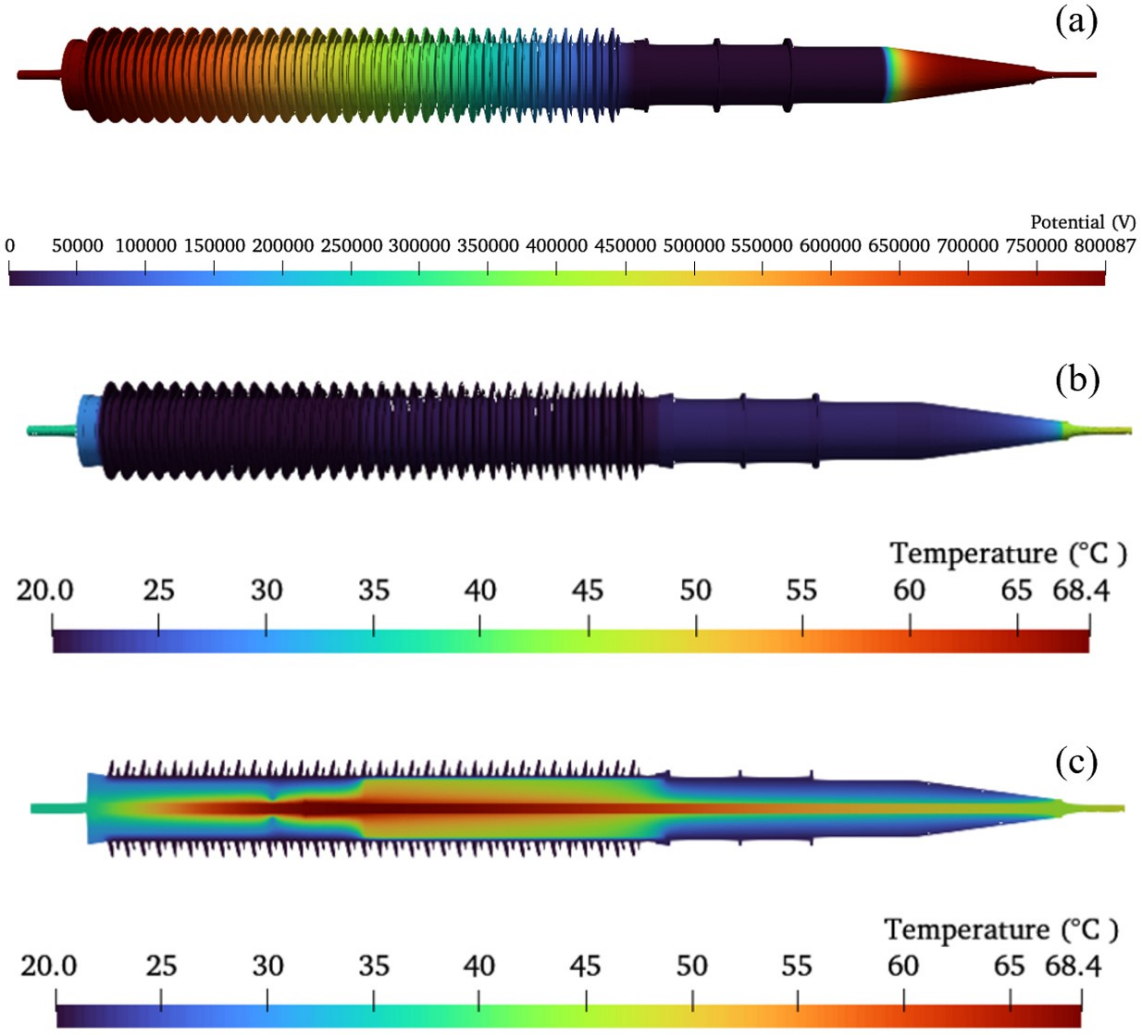
Calculation results of electro-thermal coupling. (a) The distribution of electric potential, (b) The distribution of temperature, (c) The temperature distribution cross-section.

Calculate the thermal-electric coupling of the bushing using Joule heating as the heat source, applying the boundary conditions shown in Fig 5. The resulting potential and temperature distributions within the 800 kV bushing are depicted in Fig 6, where Fig 6(c) represents a cross-sectional temperature distribution along the central axis of the bushing.

**Fig 7:**
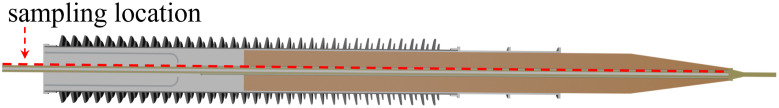
Sampling location.

**Fig 8:**
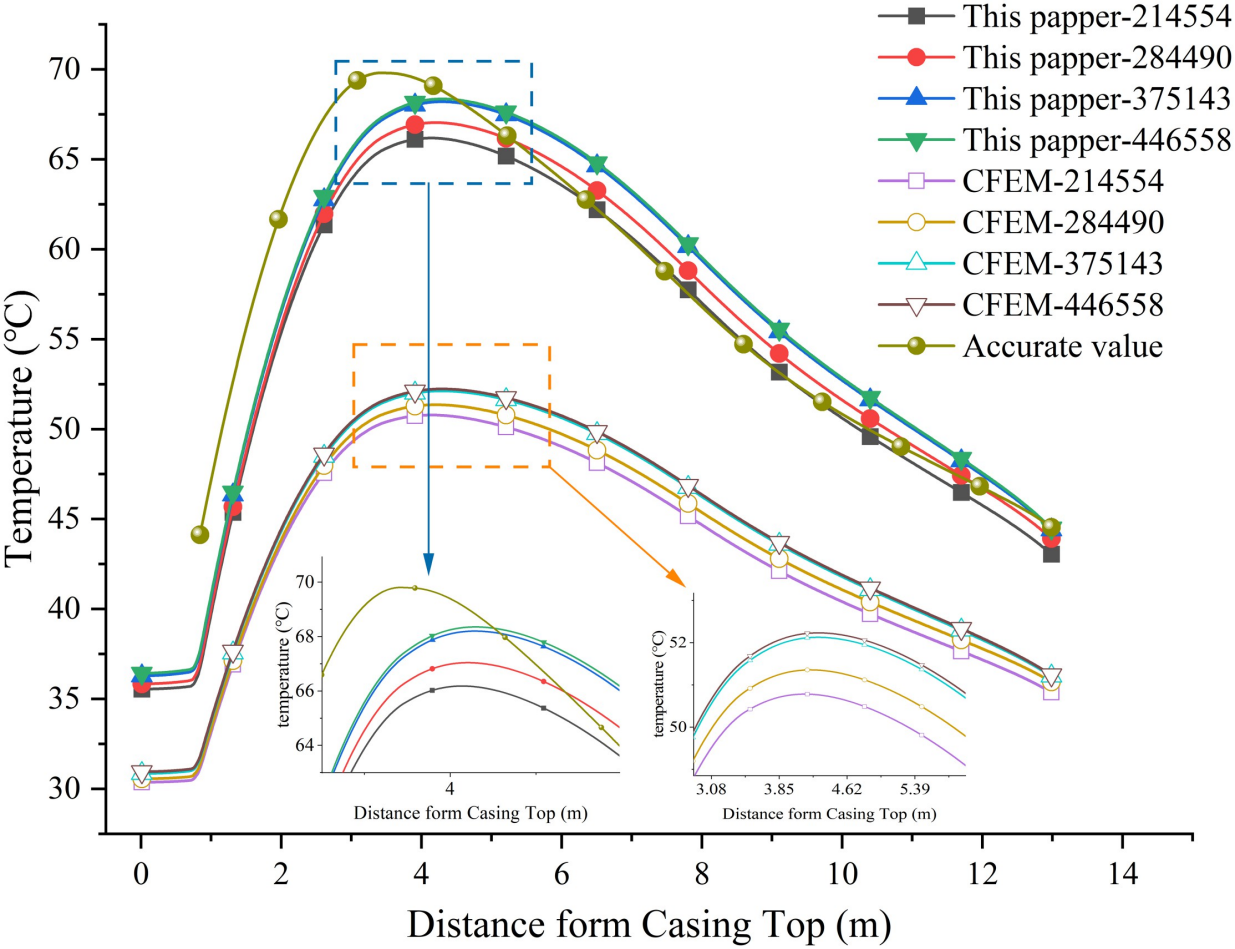
Temperature on characteristic lines.

Using the axial profile along the surface of the bushing conductor as the sampling location, temperature data is extracted. A comparison of computational accuracy between unbiased and smoothly interpolated finite element methods (FEM) with varying node counts is conducted. The sampling locations are illustrated in Fig 7, and the corresponding temperatures at these locations are depicted in Fig 8.

As shown in Fig 8, it can be observed that both the unbiased interpolated finite element method and CFEM approach closer to the accurate solution as the number of nodes increases. Under the same node count, the results obtained from the unbiased interpolated finite element method are closer to the accurate solution compared to CFEM.

**Fig 9:**
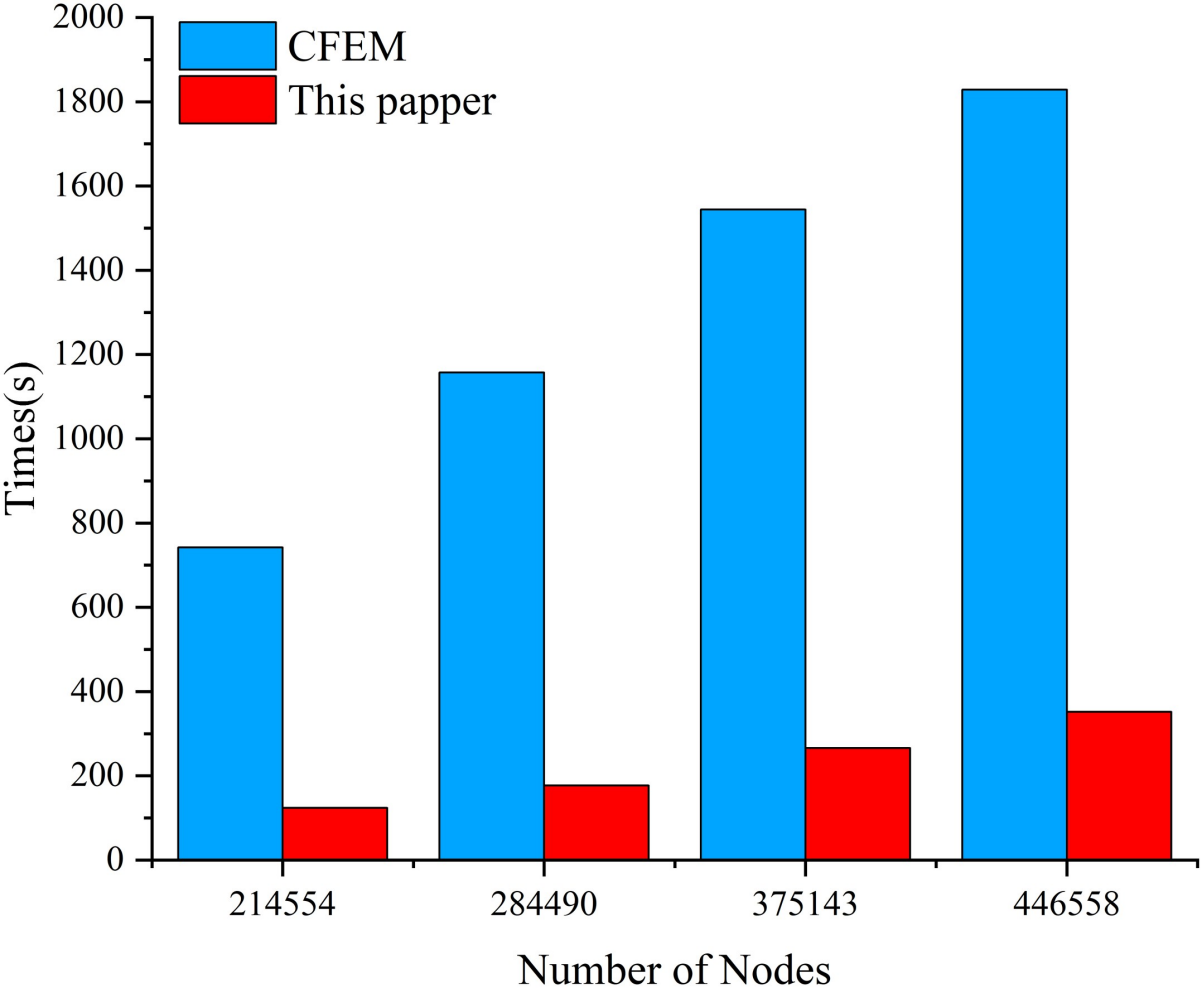
Comparison of calculation speed between SFEM and CFEM using different number of nodes.

The computation times for both algorithms at different numbers of nodes using a first-order mesh are recorded in Fig 9. It can be observed that as the mesh is refined, the solution times for both algorithms increase to varying degrees. The solution time for traditional finite element method (CFEM) increases rapidly with an increase in the number of nodes. SFEM, on the other hand, has a significantly shorter solution time compared to CFEM. With the mesh settings used in this study, the maximum computation time differs by a factor of 6.54. This indicates that, while maintaining the same level of accuracy, using SFEM can effectively improve the computational speed of electro-thermal coupling equations. It is also well-suited for the calculation requirements of power equipment with large curved structures, such as bushing insulators.

Furthermore, using the electric-thermal coupling model for dry-type bushings established in this paper, we will discuss the impact of typical bushing faults (poor contact, air temperature, and oil temperature) on bushing heating.

(1) Impact of contact resistance on Bushing Heating

**Fig 10:**
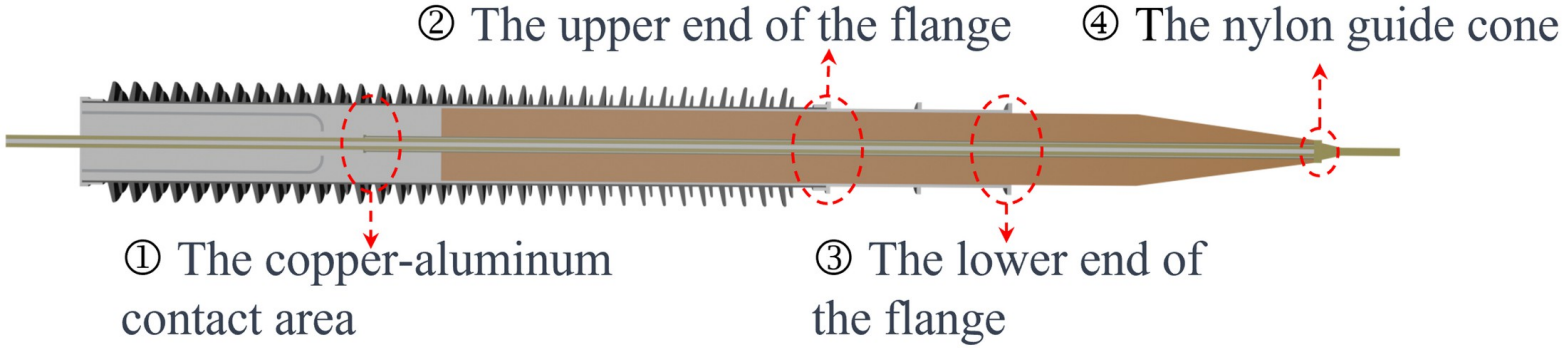
Typical fault locations of contact resistance.

In accordance with on-site operational conditions, faults resulting from abnormal contact resistance at four critical locations within the bushing are simulated. These locations include the upper flange, lower flange, copper-aluminum contact point, and nylon guide cone, as depicted in the Fig 10.

Set the simulation parameters as shown in the Table 3.

**Table 3:** Simulation parameter settings.

parameter	value
ambient temperature	50°C
Oil temperature	90–110°C
Contact resistance	1×10^−6^~2×10^−5^

**Fig 11:**
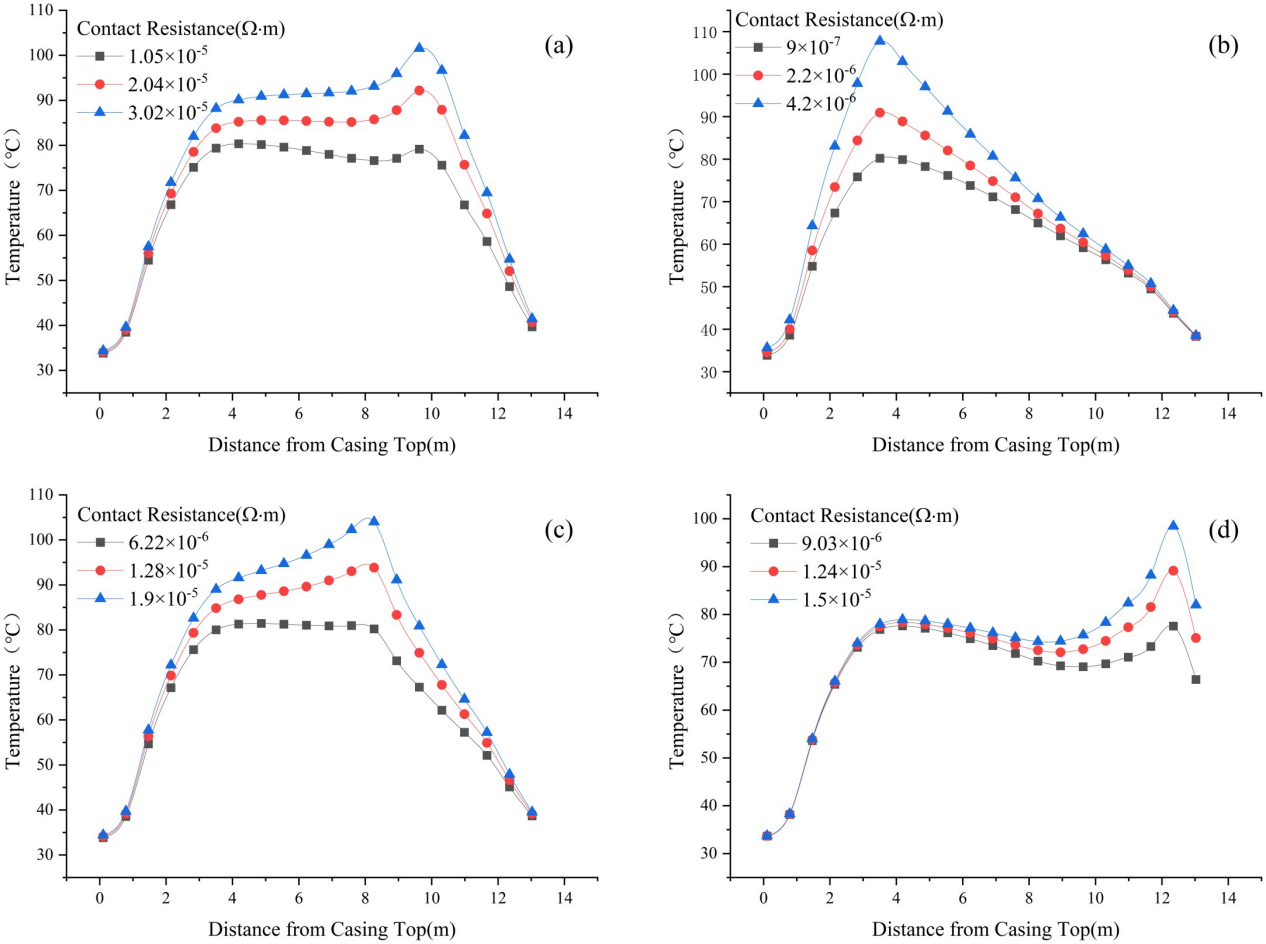
Temperature profiles along axial direction for contact resistance at different locations: (a) flange lower end, (b) copper-aluminum contact, (c) flange upper end, (d) nylon guide cone.

Fig 11 displays temperature sampling values corresponding to three different values of contact resistance at four typical locations. Specifically, Fig 11(a) to 11(d) correspond to the upper end of the flange, the copper-aluminum contact point inside the conductor, the lower end of the flange, and the guide cone, respectively.

Based on the temperature variation trends observed in Fig 11(a) to 11(d), the following conclusions can be drawn:

The degradation of contact resistance and the nearby temperatures and temperature gradients tend to increase. Among the four typical locations, the degradation of contact resistance at the copper-aluminum contact point and the nylon guide cone has the most significant impact on temperature gradients. Therefore, these areas, specifically the copper-aluminum contact point and the nylon guide cone, should be the primary focus for maintenance and equipment production due to their critical influence on temperature gradients.

(2) Impact of Ambient Temperature on Bushing Heating

Due to the changes in seasons and the day-night cycle, outdoor temperature can vary significantly, especially in high-altitude areas, and this variation has the potential to impact indoor temperatures. Additionally, temperature control systems do not maintain a constant temperature in the valve hall but allow it to fluctuate within a certain range. Therefore, it is essential to analyze the relationship between environmental temperature and the distribution of temperature fields within the bushing.

**Fig 12:**
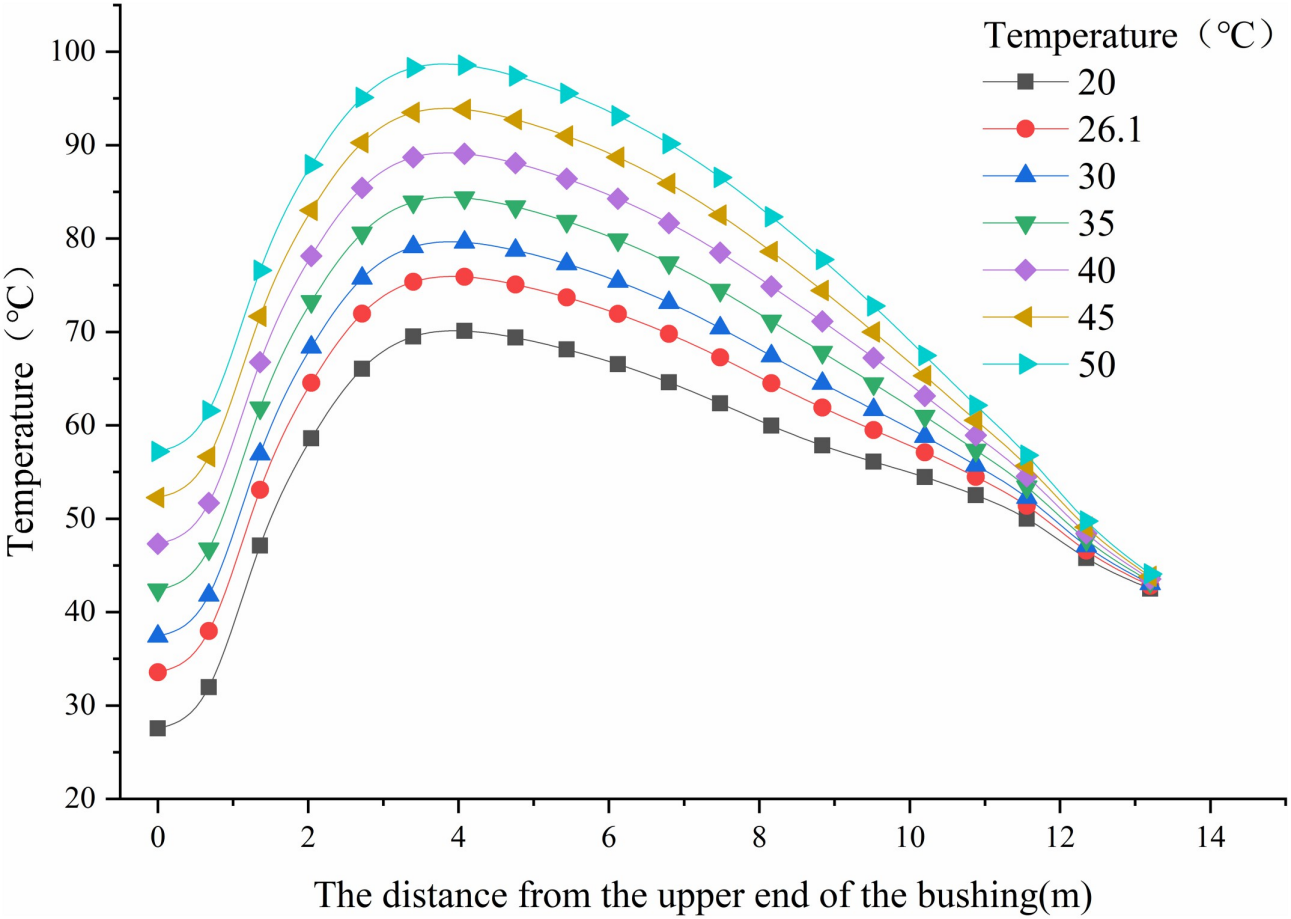
Influence of different ambient temperatures on temperature distribution of conducting rod at oil temperature of 29°C.

In the simulation model, the environmental temperature has been set at multiple different temperature points: 20°C, 26.1°C (simulating normal operational conditions on-site), 30°C, 35°C, 40°C, 45°C, and 50°C. This variation is introduced to investigate the impact of environmental temperature on the heating of the bushing. In this simulation, the oil temperature is maintained at 29°C, and heat boundary conditions for the insulating oil are set as shown in Fig 5. The computed results at the locations indicated in Fig 7 for each simulated temperature are extracted and presented in Fig 12.

From Fig 12, it is evident that under the influence of different environmental temperatures, the highest temperature is observed at the copper-aluminum contact point. Due to the impact of contact resistance, the copper-aluminum contact point is the most affected by environmental conditions. The temperature rise percentage at this location may exceed 150% compared to the temperature during normal on-site operation. This could significantly impair or disrupt the normal functioning of the equipment.

(3) Influence of Oil Temperature on Bushing Heating

While there are strict control standards for the oil temperature inside the transformer tank, its temperature control is still limited within a certain temperature range, and temperature fluctuations can potentially affect the temperature distribution of the bushing. Additionally, during actual operation, the temperature control system of the transformer tank may experience malfunctions, necessitating an assessment of the bushing’s tolerance to different temperature control system failures.

**Fig 13:**
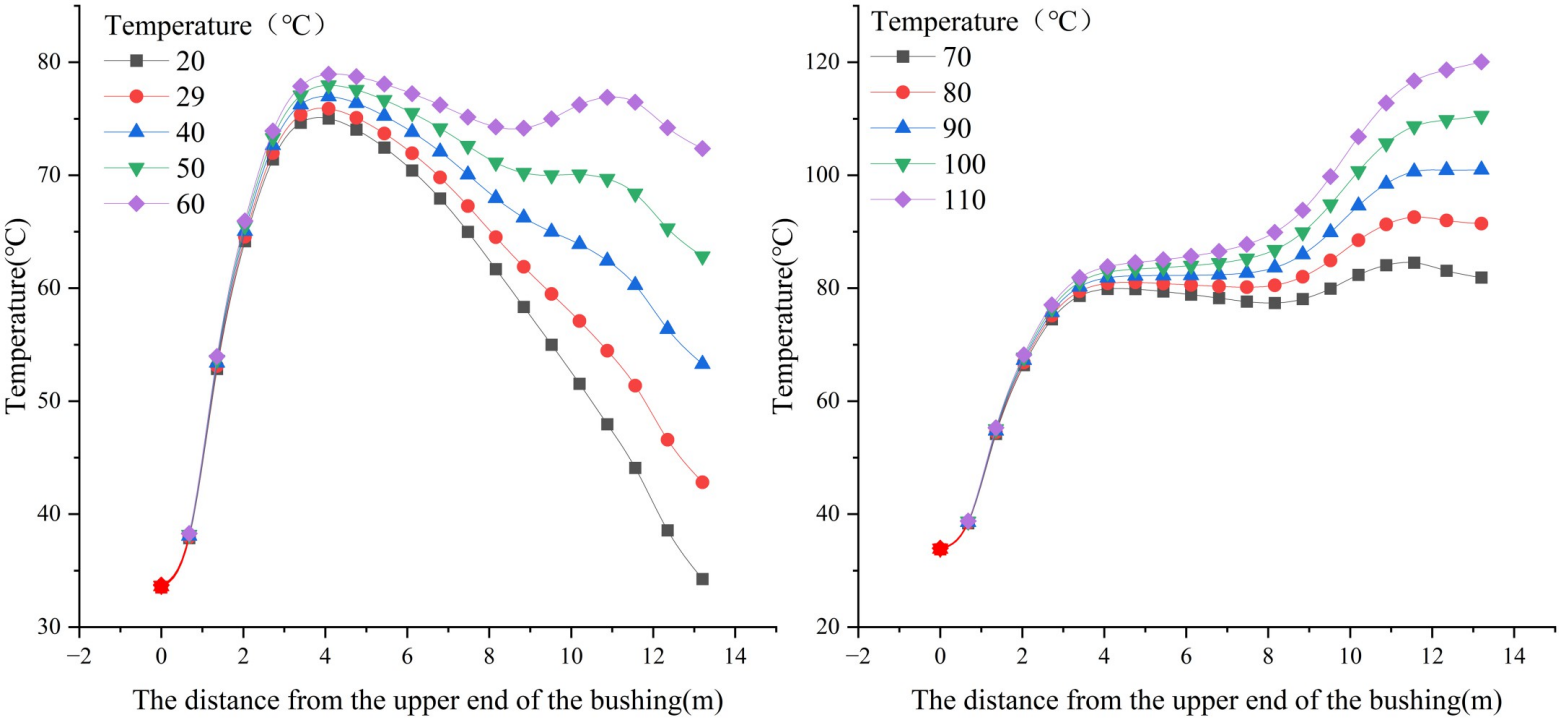
Influence of different oil temperatures on temperature distribution of conducting rod.

Therefore, it is essential to investigate the influence of oil temperature on the temperature field distribution in the transformer bushing. Fig 13 shows the temperature variation curves at the sampling locations when the oil temperature ranges from 20°C to 110°C.

From Fig 13, it is evident that under the influence of different oil temperatures, the temperature distribution along the conductor rod varies. At lower oil temperatures (≤60°C), the temperature variation is most pronounced at the copper-aluminum contact point. Conversely, at higher oil temperatures (>60°C), the temperature variation is most significant at the nylon guide cone location. In particular, the nylon guide cone is more sensitive to oil temperature fluctuations, with the temperature rise percentage exceeding 100% compared to the temperature during normal operation.

## Conclusion

This paper presents a high-precision electric-thermal coupling solution method based on the body-smoothed finite element method. The method involves dividing grid elements into multiple smooth domains and derives the integral form of electric-thermal coupling equations based on these smooth domains. It provides simplified unit discrete equations using smooth functions. The primary innovations are concentrated in the integral process of the algorithm:

1. This study employs the division of smooth domains and approximates field strength and heat flux based on these smooth domains.
2. The shape function integrals are simplified into algebraic forms, obtaining the electric and thermal conduction matrices in cases without biased derivatives.

Finally, using high-voltage dry-type bushings as an example, the improved SFEM established in this paper is used to study typical temperature anomalies in the bushings. It demonstrates that this algorithm maintains the same overall coupling equation structure. The new algorithm is easily integrated into existing computational frameworks and can be used to optimize the computational process of multiple physical fields in a similar manner, transforming the grid-based computation into a smooth domain-based computation. This approach further advances the application of simulations in the field of power equipment condition assessment, particularly in terms of computational efficiency.
